# Assessment of SARS-CoV-2 Reinfection 1 Year After Primary Infection in a Population in Lombardy, Italy

**DOI:** 10.1001/jamainternmed.2021.2959

**Published:** 2021-05-28

**Authors:** Josè Vitale, Nicola Mumoli, Pierangelo Clerici, Massimo De Paschale, Isabella Evangelista, Marco Cei, Antonino Mazzone

**Affiliations:** 1 Magenta Hospital, ASST Ovest Milanese, Magenta, Italy; 2 Legnano Hospital, ASST Ovest Milanese, Legnano, Italy

## Abstract

This cohort study examines the rate of SARS-CoV-2 reinfection among people in Lombardy, Italy, who previously recovered from COVID-19.

Despite more than 150 million people becoming infected worldwide, SARS-CoV-2 reinfections are uncommon. The risk of a second infection in the population who has recovered from COVID-19 is crucial to improve quarantine management and optimize the ongoing vaccination campaign. The rate of reinfection among health care workers has been reported,^1,2^ but the rate of reinfection in the general population is less clear.^3,4^

## Methods

We investigated the incidence of SARS-CoV-2 primary infection and reinfection among individuals who, during the first wave of the pandemic in Italy (February to July 2020), underwent diagnostic reverse-transcriptase–polymerase chain reaction (PCR; see eAppendix in the Supplement for the platform and specifics). Symptomatic and asymptomatic patients of any age, who were recruited in several screening and contact-tracing programs, were included. We obtained the approval of the local ethics committee, which, because of the observational characteristic of the study, granted a waiver of informed consent for participants. This study followed the Strengthening the Reporting of Observational Studies in Epidemiology (STROBE) reporting guideline for cohort studies.

The study laboratory serves 4 hospitals (1400 beds) and one of the most severely affected sanitarian areas (560 Kmq; 470 000 inhabitants) in Lombardy, Italy, yielding 122 007 PCR test results. We defined cases (those with infection who were PCR-positive) and controls (those without infection who were PCR-negative) according to the World Health Organization guidelines; criteria are specified in eAppendix in the Supplement. The cohorts were considered to be at risk from the time of the first definition (date of positive test result for cases; date of second negative test result for controls) until the end of the observation (February 28, 2021) or a new positive PCR test result. Reinfections were defined by a second RT-PCR positivity beyond 90 days after complete resolution of the first infection and with at least 2 consecutive negative test results between episodes.^5^ The 90-day window was decided on the basis of reports of RNA virus persistence until 12 weeks.^5^ Statistical analyses were conducted using JMP, version 14.0 (SAS Institute), and Prism, version 9.0.2 (GraphPad). Statistical significance was set at *P *< .05.

## Results

The baseline demographic characteristics are shown in the Table. The median (interquartile range) age of the patients was 59 (40-78) years, but positive cases were older and geographically distributed more in the industrial area of Legnano.

**Table.** Population Characteristics

Characteristic	SARS-CoV-2 RT-PCR		
	Negative result at baseline and during follow-up (n = 12 968)	Negative result that converted to positive during follow-up (n = 528)	Positive result at baseline (n = 1579)
Age, y			
Mean (SD)	57 (23)	58 (23)	62 (18)
Median (IQR)	59 (40-77)	59 (41-78)	63 (50-78)
Range	0-108	0-100	0-107
Sex, No. (%)			
Women	6960 (53.7)	315 (59.7)	771 (48.8)
Men	6008 (46.3)	213 (40.3)	808 (51.2)
Racial/ethnic group, No. (%)			
White	11 390 (87.8)	494 (93.6)	1449 (91.8)
Asian	578 (4.5)	15 (2.8)	41 (2.6)
Black	466 (3.6)	7 (1.3)	22 (1.4)
Latinx	506 (3.9)	12 (2.3)	59 (3.7)
Other	28 (0.2)	0	8 (0.5)
Health district, No. (%)			
Legnano	7441 (57.4)	293 (55.4)	798 (50.5)
Magenta	4203 (32.4)	192 (36.4)	728 (46.1)
Abbiategrasso	737 (5.7)	20 (3.8)	47 (3.0)
Cuggiono	587 (4.5)	23 (4.4)	6 (0.4)
No. of tests, median (IQR)	3 (3-4)	4 (4-5)	3 (3-5)
Person-day of follow-up	3 499 503	112 974	496 586
Inpatients, No. (%)	3547 (27.4)	308 (58.3)	1176 (74.5)
Symptomatic, No (%)	5554 (42.8)	371 (70.3)	1105 (70.0)

Abbreviations: IQR, interquartile range; RT-PCR, reverse-transcription–polymerase chain reaction.

During the follow-up (mean [SD], 280 [41] days) 5 reinfections (0.31%; 95% CI, 0.03%-0.58%) were confirmed in the cohort of 1579 positive patients. Most of these patients were evaluated, treated, and followed in hospitals or dedicated COVID-19 ambulatories.^6^ Only 1 was hospitalized, and 4 patients had a close relationship (2 patients work in hospitals, 1 patient underwent transfusions every week, and 1 patient retired in a nursing home) with health facilities. The mean (SD) interval between primary infection and reinfection was longer than 230 (90) days.

Of 13 496 persons who initially were not infected with SARS-CoV-2, 528 (3.9%; 95% CI, 3.5%-4.2%) subsequently developed a primary infection. The incidence density per 100 000 person days was 1.0 (95% CI, 0.5-1.5) for reinfections compared with 15.1 (95% CI, 14.5-15.7) for new infections, while the incidence rate ratio adjusted for age, sex, ethnicity, and the sanitarian area was 0.07 (95% CI, 0.06-0.08). After analyzing the cumulative incidence during follow-up, we confirmed that the 2 cohorts were significantly different (hazard ratio, 0.06; 95% CI, 0.05-0.08; log-rank test *P* < .001) (Figure).

**Figure.**
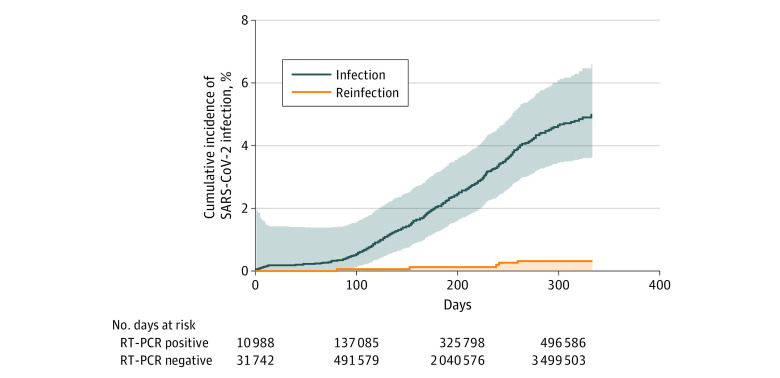
Cumulative Incidence of SARS-Cov-2 Infection RT-PCR indicates reverse-transcriptase–polymerase chain reaction.

## Discussion

The study results suggest that reinfections are rare events and patients who have recovered from COVID-19 have a lower risk of reinfection. Natural immunity to SARS-CoV-2 appears to confer a protective effect for at least a year, which is similar to the protection reported in recent vaccine studies. However, the observation ended before SARS-CoV-2 variants began to spread, and it is unknown how well natural immunity to the wild-type virus will protect against variants.

## Notes

Supplement. eAppendix.
