# Supplementary material for: Assessment of SARS-CoV-2 Reinfection 1 Year After Primary Infection in a Population in Lombardy, Italy
Source: JAMA Intern Med. 2021 May 28;181(10):1407–8. doi: 10.1001/jamainternmed.2021.2959 (PMC8164145; doi:10.1001/jamainternmed.2021.2959)
Supplement: Supplement. — eAppendix. [file jamainternmed-e212959-s001.pdf]

Supplemental Online Content

Vitale J, Mumoli N, Clerici P, et al. Assessment of SARS-CoV-2 reinfection 1 year after primary infection in a population in Lombardy, Italy. *JAMA Intern Med*. Published online May 28, 2021. doi:10.1001/jamainternmed.2021.2959

eAppendix.

This supplemental material has been provided by the authors to give readers additional information about their work.

© 2021 Vitale J et al. *JAMA Internal Medicine.*

eAppendix

Cohort Allocation Criteria

a. Case:

We considered confirmed SARS-CoV-2 infections all the patients with positive PCR if they were symptomatic or contact of a probable case, and with a cycle threshold (Ct)<35.

Low positive cases (Ct ≥ 35) needed a second concordant control within seven days to be considered as positive. If the second control was negative it needed another test to confirm final allocation.

b. Control:

Due to the low sensitivity of PCR test, the cohort of not infected was defined by two consecutive negative results.

If any negative control, symptomatic or contact of a probable case, became positive within 3 weeks from the last negative PCR-test it switched in the positive cohort of cases.

PCR platforms

RT-PCR was performed using four commercial assays:

1. Allplex TM SARS-CoV-2 Assay (targeting E, N, and RdRp/S genes; Seegene Inc., Seoul, Korea) (on CSF96 instrument, Bio-Rad Laboratories, Hercules, CA, USA)
2. GeneFinder COVID-19 Plus RealAmp Kit (targeting N, RdRp, and E genes; Osang Healthcare, Anyang-si, Korea) (on Elite InGenius instrument, ELITech Group, Turin, Italy)
3. Simplexa TM COVID-19 Direct (targeting ORF 1ab and S genes; DiaSorin Molecular LLC, Cypress, CA, USA) (on LIAISON MDX instrument, LLC, Cypress, CA, USA)
4. Xpert ®Xpress SARS-CoV-2 (targeting E and N2 genes; Cepheid, Sunnyvale, California, USA) (on GeneXpert instrument, Cepheid, Sunnyvale, California, USA).

Statistical Analysis

Each individual with a PCR test was followed up from the time of their first allocation, irrespective of the date and whether they had a positive or negative result, until Feb 28, 2021, or a new positive test, whichever first occurred. Reinfections were defined by CDC as a second RT-PCR positivity beyond 90 days, after complete resolution of the first infection and with at least two consecutive negative tests between episodes. The 90-day window was decided on the basis of reports of RNA virus persistence until 12 weeks.

We analyzed the cumulative incidence using the total number of considered individuals in each cohort and incidence density using the total person-time at risk. We compared the infection rate of cases (reinfections), observed during follow-up, with the rate observed of the controls (primary infections). We estimated the adjusted rate ratio (RR) using Poisson regression, adjusted for sex, age group (0–17, 18–64, 65–84, and ≥85 years), ethnicity and sanitarian area (Legnano, Magenta, Abbiategrasso,

© 2021 Vitale J et al. *JAMA Internal Medicine.*

Cuggiono) to control potential confounding.

We plotted a Kaplan-Meier curve of cumulative incidence of infection in the two cohorts. We report estimates using 95% CIs. We did all analyses using JMP (version 14.0) and GraphPad Prism (version 9.0.2).

© 2021 Vitale J et al. *JAMA Internal Medicine.*
